# “Salesmanship in Dentistry”: A Critical Inquiry

**Published:** 1921-05

**Authors:** Edward C. Kirk


### "SALESMANSHIP IN DENTISTRY": A CRITICAL INQUIRY

BY EDWARD C. KIRK, D.D.S., SC.D.

When the dental profession a decade ago entered upon its campaign of oral hygiene propaganda, the object of which was to popularize dentistry to the end that the public might be educated to the importance of mouth hygiene and the care of the teeth as a general health measure, there were those in the profession who sounded a note of warning that the profession would be accused of personal selfish ends by such a movement, that the dental profession was not prepared to efficiently meet any greatly increased demand for dental service, and that we were seeking responsibilities which we were ill prepared to assume.

A retrospective view of the situation is all too convincing that each one of these prophecies has been fulfilled. We have succeeded far beyond our anticipations in creating an enormously increased demand for dental service which we are not yet prepared to meet. We have had thrown upon us, by the medical profession and the public, responsibilities which we are ill prepared to assume; and last, but not least, we have painful evidences of selfish motives in connection with oral hygiene propaganda, expressing themselves definitely and nauseously in efforts to commercialize this increased demand for dental services.

One of the "nervous reflexes" of the recent world war which seems to have found expression in dentistry is that of monetary greed, justified in the minds of some by the altered world conditions which seem to have encouraged money madness, to say nothing of profiteering. Distinct evidence of this mental attitude is seen in dentistry in the increasing talk and writings on efficiency methods and "salesmanship in dentistry." We do not deprecate the laudable effort on the part of the professional man to increase his efficiency, and we believe that much improvement can very profitably be made in this direction, but we are fearful that this striving for efficiency to the *n*th power coupled with the demoralizing effect of the salesmanship *motif* will becloud the primary ideal of dentistry, and that the dollar mark will assume an inordinate position in our profession.

Dentistry is a humanitarian profession; its devotees are supposed to have chosen the profession primarily from humanitarian motives and incidentally as a means of livelihood. As a means of livelihood, the dentist is entitled to and should receive a fee for his service commensurate with its value to the individual, but to place his service on the commercial basis of selling it to the highest bidder and devising co-operative schemes to raise the bid is unworthy of the professional man.

No person has rightfully a place in a profession that he does not love. The mere fact that he has fulfilled the requirements of the law to practice, and that protects him in his practice, does not necessarily mean that he has the proper spirit toward and love of his profession or that its ideals can be safely entrusted to his care. It is far more essential that he possess the love for the sciences and associated labor of his profession and the spirit of altruism that will impel him to assume his full duty in the service of his *clientele* and as a contributor to the scientific progress and advancement of his profession.

Altruism should be the animating principle of the professions. We can not selfishly live for self alone. We share the enjoyments, pleasures and benefits resulting from the labors of others and so it becomes our duty likewise to contribute our quota to the sum of human knowledge, public welfare and world progress. Especially is this incumbent upon the members of a profession whose duty to humanity is discharged in accordance with the opportunities which it affords and in the true ethical spirit.

Professional men are notoriously poor business men, and there is not the least doubt that every practitioner owes it to himself and those dependent upon him to obtain sufficient business training to enable him to conduct his practice on sound business principles; but such training should be available through the professional college course, and not be taught from the point of view of present-day business principles as applied to commerce, as such means unduly emphasized overshadow and obscure the real mission of his profession.

We read and hear so much of efficiency lectures and so-called salesmanship in dentistry that we are constrained to enjoin "those who have ears to hear, let them stuff them with cotton" lest we be led after false gods in our eagerness to become modern salesmen. Efficiency methods applied to dental practice certainly enable one to serve more patients and in the least amount of time, but efficiency methods applied to the treatment of human ailments tend to the establishment of the same procedure for each case, which savors much of an attempt to "measure the workings of the Almighty with the yardstick" and develops a routine which must in the end lose sight of individuality. Efficiency developed to the ends of service is one thing; efficiency as a means to get money is quite another.

Let us call a halt and ponder with veneration the animating ideals of those whose efforts are responsible for the high position that dentistry occupies today. Did they seek to become proficient in salesmanship in order to sell their services to the highest bidder? Did they seek to commercialize the oral misfortunes of humanity? Did they commercialize their individual knowledge by offering it to their fellow practitioners in private classes at so much per course? Did they charge dental societies large fees for presenting papers and clinics to their members?

The long list of beacon lights of dental history who have spent their declining years in strained circumstances most eloquently answers the question and is a glowing tribute to their unselfish sacrifice for those who were to follow them. With such a heritage from those we love and honor can we afford to risk the charge of attempting to commercialize the heritage we received from those who gave their all for the pure love of their profession?

We do not wish to be considered hypercritical or hysterical alarmists, but we do believe there is cause for alarm in the growing tendency to commercialism in a profession which is avowedly dedicated to the health service of humanity.

The dental profession is more prominently in the limelight of publicity than ever before; we are in a sense passing through the test of fire and, if we fail to meet the responsibilities placed upon us, we are certain to subject ourselves to a moral castigation such as that which Hunter pronounced upon our mechanical methods.

Let us not justify the criticism that the health propaganda which we have thus far so successfully conducted was for selfish purposes, and to that end let us fight this growing tendency to commercialism with the same degree of earnestness that we protect our professional honor.—*Dental Cosmos.*

